# iBLP: An XGBoost-Based Predictor for Identifying Bioluminescent Proteins

**DOI:** 10.1155/2021/6664362

**Published:** 2021-01-07

**Authors:** Dan Zhang, Hua-Dong Chen, Hasan Zulfiqar, Shi-Shi Yuan, Qin-Lai Huang, Zhao-Yue Zhang, Ke-Jun Deng

**Affiliations:** ^1^School of Life Science and Technology and Center for Informational Biology, University of Electronic Science and Technology of China, Chengdu 610054, China; ^2^Key Laboratory of Medical Bioinformatics, Key Laboratory of Ministry of Education for Gastrointestinal Cancer, School of Basic Medical Sciences, Fujian Medical University, Fuzhou 350001, China

## Abstract

Bioluminescent proteins (BLPs) are a class of proteins that widely distributed in many living organisms with various mechanisms of light emission including bioluminescence and chemiluminescence from luminous organisms. Bioluminescence has been commonly used in various analytical research methods of cellular processes, such as gene expression analysis, drug discovery, cellular imaging, and toxicity determination. However, the identification of bioluminescent proteins is challenging as they share poor sequence similarities among them. In this paper, we briefly reviewed the development of the computational identification of BLPs and subsequently proposed a novel predicting framework for identifying BLPs based on eXtreme gradient boosting algorithm (XGBoost) and using sequence-derived features. To train the models, we collected BLP data from bacteria, eukaryote, and archaea. Then, for getting more effective prediction models, we examined the performances of different feature extraction methods and their combinations as well as classification algorithms. Finally, based on the optimal model, a novel predictor named iBLP was constructed to identify BLPs. The robustness of iBLP has been proved by experiments on training and independent datasets. Comparison with other published method further demonstrated that the proposed method is powerful and could provide good performance for BLP identification. The webserver and software package for BLP identification are freely available at http://lin-group.cn/server/iBLP.

## 1. Introduction

It is common to produce and send out visible lights in some living organisms, for example, ctenophora, bacteria, annelids, fungi, fish, insects, algae, and archaea [1]. These phenomena can be explained with mainly two mechanisms, bioluminescence and chemiluminescence, in which the former involves a series of chemical reactions, and the latter is related to absorption of light from external sources and its emission after transformation [2]. In particular, bioluminescent proteins (BLPs) play a critical role in the bioluminescence as they can convert energy released by a chemical reaction into light emission within living organisms [3]. Besides, luciferin and luciferase are two kinds of essential chemicals in the bioluminescence process. In the presence of oxygen, the luciferase, acting as an enzyme, can catalyze and speed the oxidation of substrate luciferin to produce light and form unstable intermediate product named oxyfluorescein. Sometimes luciferin and luciferase, as well as cofactor such as oxygen, are combined together in a single unit to be a stable protein complex, photoprotein, that can be triggered to emission light when mediated by cofactors such as calcium ions or ATP [4]. Furthermore, the color of the light emission can be designed by several factors like the predominant environment of bioluminescent organisms or the structure of luciferin or the amino acid sequence of the luciferase or the presence of accessory proteins such as yellow fluorescent proteins (YFPs) and green fluorescent proteins (GFPs) [5].

Bioluminescence serves various known functions, including camouflage, finding food, attraction of prey, attraction of mates, communication between bioluminescent bacteria (quorum sensing), and burglar alarm [6, 7]. Bioluminescent proteins serve as highly sensitive labels and have been widely used as invaluable biochemical tools with applications in a variety of fields including gene expression analysis, drug discovery, the study of protein dynamics and mapping signal transduction pathways, bioluminescent imaging, toxicity determination, DNA sequencing studies, and estimating metal ions such as calcium [3, 8]. Hence, identification of BLPs could help to discover many still unknown functions and promise great possibilities for medical and commercial advances. Despite BLPs can be investigated through wet-experimental methods, these methods are usually labor-intensive and time-consuming. Moreover, for most bioluminescence signals, they are too weak to detect or they are sensitive to the microenvironment, like D-luciferin, which presents different colors of light in various pH conditions [9]. As claimed in previous work [10], identification of BLPs by traditional alignment-based method like PSI-BLAST is a hard task due to poor sequence similarities among them. Thus, it is necessary to develop machine learning methods for identifying BLPs, which may provide fast and automatic annotations for candidate BLPs.

Recently, several computational methods have been proposed for predicting BLPs. The first computational method to predict BLPs is called BLProt developed by Kandaswamy et al. [10] in 2011, which was developed based on support vector machine (SVM) and 544 physicochemical properties. Soon after that, Zhao et al. [11] developed another computational method, called BLPre, by integrating position-specific scoring matrix (PSSM) and auto covariance (AC) transformation into feature vector and using SVM classifier to perform the prediction. In 2013, Fan and Li [12] published a SVM-based model by combining increment of diversity (ID) with four representative features, namely, dipeptide composition (DC), reduced amino acid alphabet (RAAA), pseudo amino acid composition PSSM (PsePSSM), and auto covariance of averaged chemical shift (acACS), to distinguish BLPs. Later, in 2014, a novel approach named SCMBLP was proposed by Huang [13] to estimate the propensity scores of 400 dipeptides and 20 amino acids based on scoring card method (SCM). In 2015, Nath and Subbiah [14] built a balanced training dataset by using unsupervised K-Means clustering algorithm and Synthetic Minority Oversampling Technique (SMOTE), then applied boosted random forest for BLP prediction. Zhang et al. [15] proposed a sequence-based method named PredBLP, which focused on sequence-derived features and adopted Fisher-Markov selector together with sequential backward selection strategy to select the optimal feature subsets. In addition to a universal model, they designed three lineage-specific classifiers, namely, bacteria, eukaryote, and archaea.

In summary, these methods mentioned above have obtained good results and provided important clues in BLP identification. However, there are still two aspects that need to be further investigated. First of all, few of them provided webservers or off-line programs and poorly maintained. Second, most of these studies only considered general BLPs, while the differences between different species of BLPs have not yet received enough attention.

**Figure 1:**
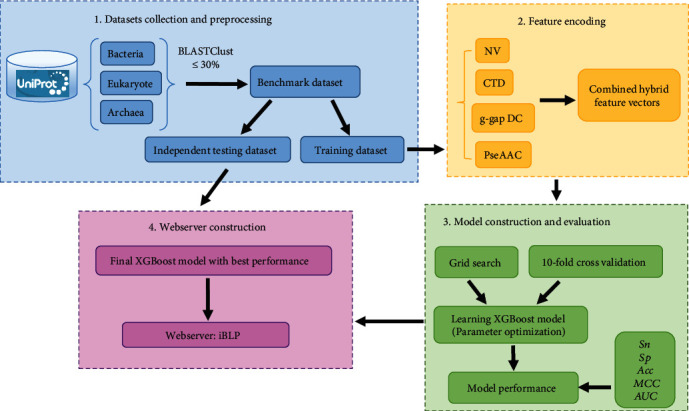
Framework of the proposed predictor iBLP to identify bioluminescent protein.

In view of the aforementioned description, in this study, we devoted to develop an ensemble tool to improve the prediction capability of BLPs. First of all, high-quality training and testing datasets were obtained. Subsequently, four kinds of feature encoding strategies were used to formulate sequence samples, including natural vector (NV), composition/transition /distribution (CTD), g-gap dipeptide composition (g-gap DC), and pseudo amino acid composition (PseAAC). Finally, our predictor was constructed based on eXtreme gradient boosting (XGBoost) classifier which was a scalable and explainable machine learning system for tree boosting. And then, based on the proposed model, a webserver named iBLP was established and available at http://lin-group.cn/server/iBLP, which could provide great assistance to the related researches. The flowchart of iBLP is shown in Figure 1.

## 2. Materials and Methods

### 2.1. Benchmark Datasets

A reliable data [16–18] is necessary for a robust model. The benchmark datasets constructed by Zhang et al. [15] were used in our work. It contained 17,403 BLPs composed of three species, namely, bacteria, eukaryote, and archaea, which were collected from UniProt (Jul. 2016). Therefore, four benchmark datasets were generated corresponding to a general and three species-specific datasets (bacteria, eukaryote, and archaea). To avoid homology bias and remove redundant sequences from the benchmark datasets, BLASTClust [19] was utilized to cluster all these protein sequences by setting the cutoff of sequence identity at 30%. And then, one protein was randomly picked from each cluster as the representative. Thus, 863 BLPs were obtained as positive samples. Among these BLPs, 748 belong to bacteria, 70 belong to eukaryote, and 45 belong to archaea. Additionally, 7093 nonredundant non-BLPs were collected to construct the negative samples that consist of 4919, 1426, and 748 proteins of bacteria, eukaryote, and archaea, respectively. Moreover, to construct balanced training dataset, 80% of the positive samples and equal number of negative samples were randomly picked out for training model. The rest positive and negative samples were used for independent testing. As a result, the final four benchmark datasets are constructed and summarized in Table 1. All data are available at http://lin-group.cn/server/iBLP/download.html.

### 2.2. Feature Encoding Schemes

#### 2.2.1. Natural Vector Method (NV)

The natural vector method (NV) was designed by Deng et al. [20] for performing evolutionary and phylogenetic analysis of biological sequence groups. Based on the natural vector method, each protein sequence can be mapped into a 60-dimensional numeric vector which contains the occurrence frequencies, the average positions, and the central moments of the twenty amino acids. This method is alignment free and needs no parameters. Thus, it has been proven to be a powerful tool for virus classification, phylogeny, and protein prediction [21–23]. Its details will be described as follows.

First, suppose that each BLP (or non-BLP) sequence sample *P* with length *L* can be formulated by

(1) $$\begin{array}{c} P=S_{1}S_{2}S_{3}\cdots S_{i}\cdots S_{L}, \end{array}$$

that is, for the set of 20 amino acids, *S*_*i*_ ∈ {*A*, *C*, *D*, ⋯, *W*, *Y*}, *i* = 1, 2, 3 ⋯ *L*. And for each of the 20 amino acids *k*, we may define

(2) $$\begin{array}{c} w_{k}\left(.\right)\text{: }\left\{A,C,D,\cdots ,W,Y\right\}\to \left\{0,1\right\}, \end{array}$$

where  *w*_*k*_(*S*_*i*_) = 1, if *S*_*i*_ = *k*. Otherwise, *w*_*k*_(*S*_*i*_) = 0.

Second, the number of amino acid *k* in the protein sequence *P*, defined as *n*_*k*_, can be calculated as follows:

(3) $$\begin{array}{c} n_{k}=\overset{L}{\underset{I=1}{\sum }}w_{k}\left(S_{i}\right). \end{array}$$

Next, let *S*_|*k*||*i*|_ be the distance from the first amino acid (regarded as origin) to the *i*-th amino acid *k* in the protein sequence, *T*_*k*_ be the total distance of each set of the 20 amino acids, and *μ*_*k*_  be the mean position of the amino acid *k*. Therefore, they can be calculated as follows:

(4) $$\begin{array}{c} \left\{\begin{array}{c} S_{\left|k\right|\left|i\right|}=i\times w_{k}\left(S_{i}\right),\\ T_{k}=\overset{n_{k}}{\underset{i=1}{\sum }}S_{\left|k\right|\left|i\right|,}\;\\ \mu_{k}=\frac{T_{k}}{n_{k}}. \end{array}\right. \end{array}$$

Let us take the amino acid sequence MCRAACGECFR as an example. For amino acid *A*, *n*_*A*_ = 2, the total distance of *A* is *T*_*A*_ = 3 + 4 = 7 since the distances from the first residue to the two *A*s are 3 and 4, respectively. Then, *μ*_*A*_ = *T*_*A*_/*n*_*A*_ = 7/2. Similarly, *T*_*C*_ = 1 + 5 + 8 = 14 with *n*_*C*_ = 3 and *μ*_*C*_ = *T*_*C*_/*n*_*C*_ = 14/3. The arithmetic mean value of total distance for other kinds of amino acids can be obtained in the same way.

Protein sequences with the different distribution of each amino acid might be different even if they have the same amino acid content and distance measurement. Therefore, the information about distribution has also been included in the natural vector. And then, the second-order normalized central moments *D*_2_^*k*^ can be defined as follows:

(5) $$\begin{array}{c} D_{2}^{k}=\overset{n_{k}}{\underset{i=1}{\sum }}\frac{{\left(S_{\left|k\right|\left|i\right|}-\mu_{k}\right)}^{2}}{n_{k}\times L}. \end{array}$$

The second normalized central moment is the variance of the distance distribution for each amino acid.

For the sufficiency annotation of protein sequences, the three groups of parameters, the number of each amino acid, the mean value of total distance of each amino acid, and the information of distance distribution, were concatenated to obtain the final natural vector. As a result, the 60-dimensional natural vector of a protein sequence *P* is obtained and defined as

(6) $$\begin{array}{c} P={\left[n_{A},\mu_{A},D_{2}^{A},\cdots ,n_{S},\mu_{S},D_{2}^{S_{i}},\cdots ,n_{Y},\mu_{Y},D_{2}^{Y}\right]}^{T}, \end{array}$$

where the symbol ^"^*T*^"^ is the transpose operator.

#### 2.2.2. Composition/Transition/Distribution (CTD)

The composition, transition, and distribution (CTD) method was first proposed for protein folding class prediction by Dubchak et al. [24] in 1995. These three descriptors composition (C), transition (T), and distribution (D) could be calculated according to the following two hypothesis: (i) the sequence of amino acids could be transformed into a sequence of certain structural or physicochemical properties of residues; (ii) according to the main clusters of the amino acid indices of Tomii and Kanehisa [25], twenty amino acids were divided into three groups based on each of the 13 different physicochemical attributes, including hydrophobicity, normalized Van der Waals volume, polarity, polarizability, charge, secondary structures, and solvent accessibility. The groups of amino acids are listed in Table 2, and the details of grouping criterion can be seen in the previous study [26]. Therefore, the three descriptors were used to describe the composition percentage of each group in the peptide sequence which could yield three features: the transition probability between two neighboring amino acids belonging to two different groups that also contained 3 features; the distribution pattern of the property along the position of sequence (the first, 25%, 50%, 75%, or 100%), which 5 features were obtained. Finally, based on the CTD method [27], a sample protein *P* can be formulated by (3 + 3 + 5) × 13 = 273 dimensional feature vector.

#### 2.2.3. g-gap Dipeptide Composition (g-gap DC)

The amino acid composition (AAC) and dipeptide composition (DC) encoding strategies have been widely used for protein prediction [28–30]. However, they can only express the fraction of each amino acid type or the adjacent sequence-order information within a protein. In fact, the interval residues in primary sequence might be spatially closer in tertiary structure, especially in some regular secondary structures, such as alpha helix and beta sheet, which are two nonadjoining residues were connected by hydrogen bonds. In other word, it means that interval residues are more significant than the adjacent residues in biology. Hence, the g-gap dipeptide composition (g-gap DC) feature encoding strategy is proposed to calculate the frequency of amino acid pairs separated by any *g* residues.

And then, a protein *P* can be formulated by

(7) $$\begin{array}{c} P={\left[f_{1}^{g},f_{2}^{g},f_{3}^{g},\cdots ,f_{i}^{g},\cdots ,f_{400}^{g}\right]}^{T}, \end{array}$$

where *f*_*i*_^*g*^ represents for the frequency of the *i*-th (*i* = 1, 2, 3, ⋯, 400) g-gap dipeptide and can be calculated by

(8) $$\begin{array}{c} f_{i}^{g}=\frac{n_{i}^{g}}{L-g-1}, \end{array}$$

where *n*_*i*_^*g*^ denotes the occurrence number of the *i*-th g-gap dipeptide and *L* is the length of protein *P*. Particularly, when *g* = 0, the g-gap DC method is equal to adjoining DC.

#### 2.2.4. Pseudo Amino Acid Composition (PseAAC)

The pseudo amino acid composition (PseAAC), proposed by Chou [31], is an efficient and widely used method to convert a protein sequence into a feature vector for developing different predictors based on machine learning algorithms [32–34]. In this work, we adopted the type-II PseAAC to represent protein samples. This method contains amino acid dipeptide composition as well as the correlation of physicochemical properties between two residues. Accordingly, each BLP (or non-BLP) sequence sample can be denoted as a 20^2^ + *nλ* dimensional vector which is formulated as follows:

(9) $$\begin{array}{c} P={\left[x_{1},x_{2},\cdots ,x_{400},x_{401},\cdots ,x_{400+n\lambda }\right]}^{T}, \end{array}$$

where *n* is the number of amino acid physicochemical properties considered, including hydrophobicity, hydrophilicity, mass, pK1, pK2, pI, rigidity, flexibility, and irreplaceability, which has been used in [35]; thus, *n* = 9 here. Since first six properties have been widely used in protein bioinformatics, we will briefly discuss the latter three properties: rigidity, flexibility, and irreplaceability. The rigidity and flexibility of amino acid side chains have been pointed out by Gottfries et al. [36] that it was a key for forming polypeptides and local protein domains associated with protein property alterations. Moreover, the rigidity and flexibility properties of sequences were used to predict conformation and protein fold changes and were verified by NMR measurement [37]. Besides, the degree of difficulty of residues' replacement is different in the evolution. Thus, the irreplaceability is a response to mutational deterioration in the course of the evolution of life [38]. The original values of nine physicochemical properties can be accessed at http://lin-group.cn/server/iBLP/download.html. *λ* represents the rank of correlation. *x*_*u*_ (*u* = 1, 2, ⋯, 400 + *nλ*) stands for the frequencies for each element and can be calculated as follows:

(10) $$\begin{array}{c} x_{u}=\left\{\begin{array}{c} \frac{f_{u}}{\sum_{i=1}^{400}f_{i}+\omega \sum_{j=1}^{9\lambda }\varphi_{j}},\left(1\leq u\leq 400\right),\\ \frac{\omega \varphi_{j}}{\sum_{i=1}^{400}f_{i}+\omega \sum_{j=1}^{9\lambda }\varphi_{j}},\left(401\leq u\leq 400+9\lambda \right), \end{array}\right. \end{array}$$

where *f*_*μ*_  represents frequency of the 400 dipeptides, *ω* is the weight factor for sequence order effect and its detailed information, and *φ*_*u*_ represents the *j*-tier sequence correlation factor of the physicochemical properties between residues. Given that this method has been commonly used and its detailed definition of more parameters could be found elsewhere [32], we do not reiterate them here.

### 2.3. eXtreme Gradient Boosting (XGBoost) Algorithm

It is well known that eXtreme gradient boosting (XGBoost) [39] is an ensemble learning algorithm based on gradient boosting and provides state-of-the-art results for many bioinformatics problems [40–42]. XGBoost is essentially an ensemble method based on gradient boosted tree. The result of the prediction is the sum of the scores predicted by *K* trees, as shown in the formula below:

(11) $$\begin{array}{c} {\hat{y}}_{i}=\overset{K}{\underset{k=1}{\sum }}f_{k}\left(x_{i}\right),f_{k}\in F, \end{array}$$

where *x*_*i*_ is *i*-th of the training sample, *f*_*k*_(*x*_*i*_) is the score for the *k*-th tree, and *F* is the space of functions containing all gradient boosted trees. The objective function could be optimized by the following formula:

(12) $$\begin{array}{c} \text{obj}\left(\theta \right)=\overset{n}{\underset{i=1}{\sum }}l\left(y_{i},{\hat{y}}_{i}\right)+\overset{K}{\underset{k=1}{\sum }}\Omega \left(f_{k}\right), \end{array}$$

where the former $\sum_{i=1}^{n}l\left(y_{i},{\hat{y}}_{i}\right)\;$ stands for a differentiable loss function that measures the fitness of model prediction ${\hat{y}}_{i}\;$ and samples of training dataset *y*_*i*_, while the latter ∑_*k*=1_^*K*^*Ω*(*f*_*k*_) represents an regularization item that punishes the complexity of the model to avoid overfitting. More detailed formulas can be seen in reference [39].

Compared with the general gradient boosting and other machine learning algorithms, XGBoost has some unique advantages. First, XGBoost performs a second-order Taylor expansion for the objective function and uses the second derivative to accelerate the convergence speed of the model while training. Thus, its embedded parallel processing allows a faster learning. Especially for large-scale datasets, the improvement of training speed is more beneficial. Second, a regularization term is added to the objective function to control the complexity of the tree to obtain a simpler model and avoid overfitting. Third, XGBoost is of high flexibility and allows users to define custom optimization objectives and evaluation criteria. Meanwhile, XGBoost classifier can handle well from imbalance training data by setting class weight and taking AUC as evaluation criteria. In summary, XGBoost is a highly flexible and scalable tree structure enhancement model in that it can handle sparse data, greatly improve algorithm speed, and reduce computational time and memory for training large-scale data.

In this study, the predictive model was implemented by a python package called XGBoost (version 1.1.1), which could be download from https://pypi.org/project/xgboost/. The parameters of XGBoost, including general parameters, booster parameters, and learning task parameters, can be optimized by grid search method with cross validation in the model training stage. The selection of XGBoost's parameters will be discussed in detail in Results and Discussions.

### 2.4. Performance Evaluation Metrics

How to objectively evaluate the predictor quality is a key point for developing a powerful predictor and estimating its potential application value for BLP prediction. Thus, the following metrics [43–46], sensitivity (Sn), specificity (Sp), overall accuracy (Acc), and Matthew's correlation coefficient (MCC), are used in our work and can be, respectively, calculated as follows:

(13) $$\begin{array}{c} \left\{\begin{array}{cc} \text{Sn}=\frac{\text{TP}}{\text{TP}+\text{FN}} & 0\leq \text{Sn}\leq 1,\\ \text{Sp}=\frac{\text{TN}}{\text{TN}+\text{FP}} & 0\leq \text{Sp}\leq 1,\\ \text{Acc}=\frac{\text{TP}+\text{TN}}{\text{TP}+\text{FP}+\text{TN}+\text{FN}} & 0\leq \text{Acc}\leq 1,\\ \text{MCC}=\frac{\text{TP}\times \text{TN}-\text{FP}\times \text{FN}}{\sqrt{\left(\text{TP}+\text{FP}\right)\left(\text{TN}+\text{FN}\right)\left(\text{TP}+\text{FN}\right)\left(\text{TN}+\text{FP}\right)}} & -1\leq \text{MCC}\leq 1, \end{array}\right. \end{array}$$

where TP, TN, FP, and FN indicate the true positives (i.e., correctly predicted as BLPs), true negatives (i.e., correctly predicted as non-BLPs), false positives (i.e., incorrectly predicted as BLPs), and false negatives (i.e., incorrectly predicted as non-BLPs), respectively. The higher the value of Acc, Sn, and Sp are, the more robust the predictor is. Moreover, a value of MCC = 1 indicates the best possible prediction while MCC = −1 indicates the worst possible prediction (or anticorrelation). MCC = 0 would be expected for a random prediction scheme.

Additionally, the receiver operating characteristic (ROC) curve [47–49] can present the model behavior of the true positive rate (TPR = sensitivity) against the false positive rate (FPR = 1 − specificity) in a visual way. The area under the ROC (AUC) is also used as performance evaluation metric in this study which can quantitatively and objectively measure the performance of the proposed method. A perfect predictor is proved to have the value of AUC = 1, and the random performance is proved to have the value of AUC = 0.5.

## 3. Results and Discussion

### 3.1. Existing Computational Methods for Identifying BLPs

Recent years, some computational methods have been developed to identify BLPs and summarized in [8]. Tables 3 and 4 presented a comprehensive review on currently available dataset and computational tools for BLP identification.  Table 3 showed that the first benchmark dataset D1 for BLP prediction was established by Kandaswamy et al. [10] and collected from Pfam database [50]. To avoid potential overestimation of the prediction performance, the CD-HIT program [51] was used to remove sequence redundancy from both positive and negative datasets by setting cutoff values of 40%. Then, Zhao et al. [11], Fan and Li [12], Huang [13], and Nath and Subbiah [14] also constructed their benchmark datasets based on the first benchmark dataset by using various ways to undersample Kandaswamy's dataset. Moreover, Zhang et al. [15] built a new benchmark dataset called D2 based on UniProt database [52] for bacteria, eukaryote, and archaea species. They used BLASTClust [19] to reduce sequence redundancy by setting the cutoff value of sequence identity less than 30%.

After getting the benchmark datasets, using effective feature representation to convert sequence samples into numerical vectors is significant for developing a powerful computational method to predict BLPs. As shown in Table 4, the sequence-derived features for all existing computational methods include physicochemical properties (PCP), amino acid composition (AAC), dipeptide composition (DC), evolutionary information, and sequence motifs. Additionally, to exclude information redundancy and improve the generalization ability of the prediction model, various feature selection strategies can be applied. Both Kandaswamy et al. [10] and Nath and Subbiah [14] used ReliefF [53] to choose useful information to construct their computational tools. Increment of diversity (ID) [54] is used to measure the similarity level of two diversity sources and reduce the dimension of feature vectors, which was proposed in Fan and Li's work [12]. And then, Zhang et al. [15] utilized Fisher-Markov selector [55] together with sequential backward selection (SBS) strategy to select optimal feature subset.

Furthermore, the classification algorithm could significantly affect the discrimination capability of a prediction model. It could be seen from Table 4 that SVM was adopted as the predominant classification algorithm by multiple tools, including BLPort [10], BLPre [11], Fan's method [12], PredBLP [15]. In addition to SVM, other scoring method and machine learning algorithms were also adopted. For example, the scoring card method (SCM) was applied in SCMBLP [13] to perform classification which is a general-purpose method by calculating propensity scores of 400 dipeptides and 20 amino acids to be the protein with the investigated function; Nath and Subbiah [14] used an ensemble learning method called Real Adaboosting Random Forest (RARF) [56] for BLP classification and prediction.

As a result, from Tables 3 and 4, we could draw several conclusions: (i) most of these methods used different way to undersample Kandaswamy's dataset [10], while the potential bias might produce in the process of sampling. (ii) Six tools for BLP prediction were listed in Table 4, of which five studies did not consider species specificity, while there was only the last one designed a model for bacteria, eukaryote, and archaea species. (iii) Most of the tools were established based on SVM classification algorithm except that SCMBLP [13] and Nath's method [14]. SVM is more suitable for small sample dataset and low dimension feature set. Once the data increases, calculation of SVM will be time and memory consuming. With the availability of large BLP dataset, it is obvious that we need to adopt high efficient parallel processing algorithm to speed and improve the ability to predict BLPs. (iv) Most of the webservers to predict BLPs did not work now. Among abovementioned predictors, only four works, namely, BLProt [10], BLPre [11], SCMBLP [13], and PredBLP [15], that can provide online services. Unfortunately, only the webserver of PredBLP is still working now. However, the webserver of PredBLP allows users to predict no more than five protein sequences at a time, which is quite inconvenient to scholars and researchers to study large-scale BLP data.

It is noteworthy that these above works have found some important features in BLPs. Huang [13] pointed out that BLPs have four characteristics based on a series of analysis of informative physicochemical properties of 20 amino acids, as follows: (1) high transfer free energy of residues from inside to the protein surface, (2) high occurrence frequency of residues in the transmembrane regions of the protein, (3) large hydrophobicity scale from the native protein structure, and (4) high Pearson correlation coefficient (*R* = 0.921) between the amino acid compositions of BLPs and integral membrane proteins. Additionally, they found that top-ranked dipeptides do not tend to cluster in a certain region, which suggested that bioluminescence is a global property of BLP sequences, not occur in specific segments. Furthermore, Zhang et al. [15] proposed that BLPs were enriched with charged residues and showed high preference with A-, R-, P-, and G-related dipeptide compared with the non-BLPs. In a word, these findings have important guidance for our research about BLPs.

### 3.2. Parameter Optimization

As we all know, n-fold cross validation is a common statistical analysis method in machine learning to train and test hyperparameters of feature extraction method and prediction model [57–59]. In our work, 10-fold cross validation and grid search method were used to confirm the optimal feature subset as well as the optimal parameters of XGBoost models based on four benchmark datasets by the highest AUC values. The reason why we used AUC values as standard is that compared with sensitivity, specificity, and overall accuracy, it could provide a more objective evaluation, especially on imbalance benchmark dataset [60].

**Figure 2:**
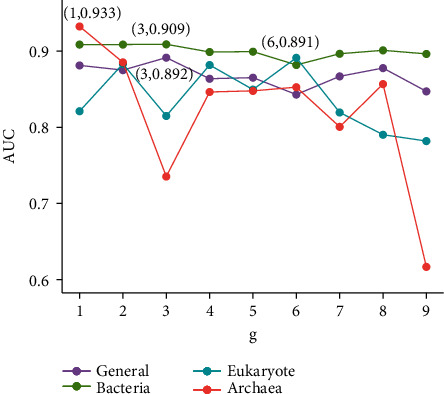
The AUCs corresponding to different *g* values on four species-specific training datasets. The peaks of AUC values of general, bacteria, eukaryote, and archaea training datasets are marked by red cubes, respectively, in 10-fold cross validation.

According to the definition in the construction of feature vector section, the information of BLP primary sequences were extracted by the integration of NV, CTD, g-gap DC, and PseAAC methods. In g-gap DC, the choice of the parameter *g* has a significant impact on the model prediction performance, which represents the correlation between any amino acid and *g* residue intervals. Additionally, for PseAAC, *λ* and *ω* also play key roles in obtaining an optimal classification model. *λ* is the correlation tier of physicochemical properties between two amino acids, which describes the global pattern sequence-order effect (long-range information) along a protein sequence; *ω* is the weight factor to adjust the ratio between short-range effect and long-range effect. As a matter of experience, the *g* values in g-gap DC method were set in the range from 1 to 9 for each training data and the performances of 9 × 4 = 36 feature subsets were input into XGBoost models and evaluated by 10-fold cross validation. The optimal *g* parameters for four species were determined by the highest values of AUC on the training dataset, as shown in Figure 2. It could be seen from Figure 2 that the highest AUCs of g-gap DC method on general, bacteria, eukaryote, and archaea training datasets are 0.892 (*g* = 3), 0.909 (*g* = 3), 0.891 (*g* = 6), and 0.933 (*g* = 1), respectively.

Since the selection of *λ* cannot exceed the length of the shortest sequence in the training dataset for PseAAC method, the *λ* and *ω* parameters were chosen through grid search method, as follows:

(14) $$\begin{array}{c} \left\{\begin{array}{c} 1\leq \lambda \leq 38,\text{with step}∆=1,\text{for general},\text{bacteria},\\ 1\leq \lambda \leq 42,\text{with step}∆=1,\text{for eukaryote},\\ 1\leq \lambda \leq 48,\text{with step}∆=1,\text{for archaea},\\ 0.1\leq \omega \leq 1,\text{with step}∆=0.1,\text{for all species}. \end{array}\right. \end{array}$$

Similarly, by examining the performances of all feature subsets for each species, we obtained the optimal parameters of *λ* and *ω* on four training datasets. The optimal parameters and dimensions of feature space according to Eq. (9) are shown as follows:

(15) $$\begin{array}{c} \text{Feature dimension of PseAAC}=\left\{\begin{array}{c} 562\text{ for general }\left(\omega =0.2,\lambda =18\right),\\ 499\text{ for bacteria }\left(\omega =0.1,\lambda =11\right),\\ 490\text{ for eukaryote }\left(\omega =1,\lambda =10\right),\\ 661\text{ for archaea }\left(\omega =0.2,\lambda =29\right). \end{array}\right. \end{array}$$

Meanwhile, the prediction models were trained and learned with the series of parameter choices and combination of XGBoost classifiers. A great deal of prior knowledge can be utilized to improve the learning process [42]; thus, the parameters which are commonly adjusted to improve the model's performance, such as n_estimators, max_depth, and learning_rate, are taken into account firstly. As a result, a set of best parameters was obtained by grid search method based on 10-fold cross validation. Therefore, the final tuning results of XGBoost models were n_estimator = 280, max depth = 12, learning rate = 0.1, and gamma = 0. Moreover, default values are adopted for other parameters.

### 3.3. Performance Evaluation on Different Features and Combinations

Through optimizing parameters, we have obtained pretrained models based on a general and three species-specific training datasets. In this section, we will investigate which features could produce the best performance on the identification of BLPs. Thus, we examined the prediction performances of 4 kinds of encoding features, namely, NV, CTD, g-gap DC, and PseAAC, using 10-fold cross validation. Moreover, to extract the feature information of protein sequences more comprehensively and realize the complementarity between different feature information, the method of feature fusion was adopted.  Table 5 recorded the details of prediction results of 4 kinds of individual feature and their combinations based on four training datasets by calculating the average values of 10 experiments. It was found that the promising prediction results have been obtained by four kinds of individual feature in which PseAAC encoding feature given out the highest AUC values of 0.900 and 0.925 for general and bacteria species, while for eukaryote and archaea species, g-gap DC method produced best performance of the highest AUC values of 0.891 and 0.838, respectively. Generally, the feature fusion might produce better prediction performance when comparing with individual features. As expected, the combination of four kinds of features for identifying BLPs achieved the best performance with *AUC* = 0.920 in general model. Although compared with individual feature, the predictive performances of combination of four features were increased slightly for other three species-specific models; they did not achieve the best predictive performance. It is well-known that noise or redundant information could reduce the model's performance, robust, and efficiency. Therefore, the phenomena about predictive performance decrease were maybe derived from information redundancy. Hence, it is necessary to explore which feature combination can produce better prediction results. As shown in Table 5, the combination of CTD, g-gap DC, and PseAAC encoding features could produce the highest AUC value of 0.936 in bacteria. For eukaryote, the combination of CTD and g-gap DC encoding features achieved the highest AUC value of 0.924. Best performance of the highest AUC value of 0.969 was obtained by the combination of NV, CTD, and g-gap DC in archaea. These results indicated that the four coding features we used were effect, and further, the combination of different kinds of features could produce a promising result.

### 3.4. Cross-Species Validation

**Figure 3:**
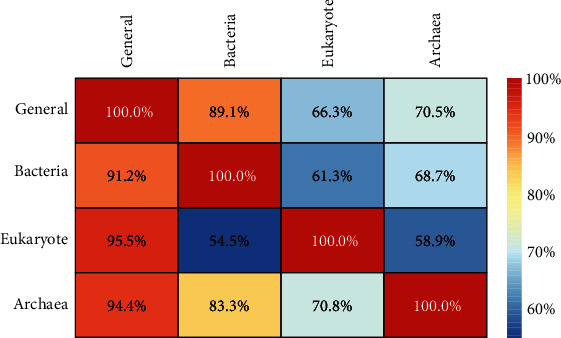
The heat map showing the cross-species prediction accuracies. Once a general or species-specific model was established on its own training dataset in columns, it was validated on the data from the all or same species as well as the independent data from the all or other three species in rows.

As stated in above section, we found that the combinations of various encoding features on general and three species-specific training datasets could produce different prediction results. It might imply that BLPs in different species have different attributes within encoding features. These inner attributes can be used to further improve the prediction performance by considering species-specific scheme. What is more, it is required to identify BLPs in more other species in addition to the species mentioned in this study. However, it may be hard due to lack of data in other species to train the models. Thus, it is necessary to demonstrate whether a model trained with the data from one species or all species (bacteria, eukaryote, and archaea in this study) could recognize the BLPs in other species. To address this confusion, we trained one general and three species-specific models using the four BLPs' training data and validated these models on the independent BLP data of other species. The predictive overall accuracies of cross-species validation were shown in Figure 3. The models in columns were tested on the other datasets in rows. As shown in Figure 3, it is obvious that the best accuracy (100.0%) was always obtained by the model built based on the data from itself. And it could be found in Figure 3 that the model constructed on general dataset achieved good results, but the models based on three specific-species datasets did not produce the desired results, which get Acc values of 89.1%, 66.3%, and 70.5% for bacteria, eukaryote, and archaea datasets, respectively. Thus, it can be concluded that the generalization ability of the model based on species-specific datasets is not strong. Moreover, the Acc values of 83.3% and 70.8% tested on archaea data are acceptable. However, it is not suitable for archaea to construct prediction model to predict bacteria and eukaryote data because the Acc values are only 68.7% and 58.9%, respectively. These experimental results indicate that the species-specific scheme and the species-specific models we established are reliable.

### 3.5. Comparison with Other Classification Algorithms

With the optimal feature combinations on four datasets, we would like to explore whether the performance of XGBoost classifier is superior to other classification algorithms based on tree model. Hence, we focus on three tree-based algorithms, i.e., decision tree, random forest, and AdaBoost. Decision tree (DT) is a nonparametric supervised machine learning method which is commonly used in data mining and classification [61, 62]. The goal of DT is to create a tree model that predicts the value of a target variable by learning simple decision rules inferred from the data features [63]. Random forest (RF) is an ensemble learning method of a large number of decision trees based on bagging. Each tree in the ensemble is trained on a subset of training instances and features that are randomly selected from the given training set. Thus, the idea of the random forest is to combine multiple weak classifiers, and then, the final decision is made by majority voting [64]. AdaBoost is a popular and powerful ensemble learning method based on boosting. It determines the weight of each sample according to whether the classification of each sample is correct in each iterative process and the overall accuracy of the last iterative process. The new training dataset with modified weights is sent to iteratively train a new weak classifier. As a result, the final decision classifier of AdaBoost is the weighted average of weak classifiers obtained from each iterative process [65].

**Figure 4:**
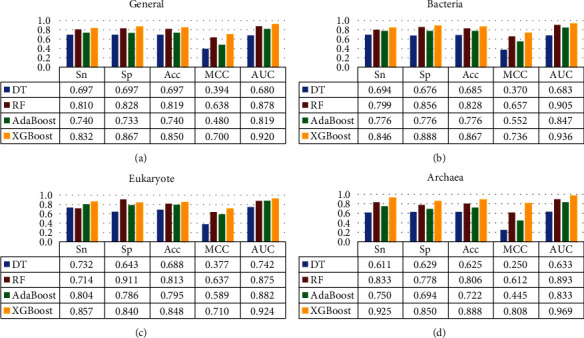
Comparison of different classification algorithms for identifying BLPs on four training datasets by 10-fold cross validation.

The abovementioned three classification algorithms were implemented by using Weka (version 3.8.3), which is a collection of machine learning algorithms for data mining tasks [66]. The default parameter values of three classification algorithms were used in Weka. Additionally, the predictive results of XGBoost classifier and the above decision tree, random forest, and AdaBoost algorithms on our four training datasets by 10-fold cross validation were plotted in Figure 4. As shown in Figure 4, decision tree classification algorithm performed worst for predicting BLPs, while XGBoost classifier could always yield best performance on four datasets. It can be concluded that the XGBoost classifier is more applicable than other three classification algorithms to identify BLPs. Therefore, the final models of the four datasets were constructed based on XGBoost algorithm.

### 3.6. Comparison with Published Methods

To further demonstrate the robust of our method, it is necessary to compare our method with other published method. Here, the PredBLP [15] was selected to perform comparison in that the same benchmark datasets were used. The results of PredBLP's method on the same training dataset by using 5-fold cross validation and independent testing dataset were directly obtained from their reports. The compared details were listed in Table 6. As shown in Table 6, although the Acc values of our method on three species-specific datasets by 5-fold cross validation are slightly lower than PredBLP's method, our predictor produced promising results with the mean AUC = 0.930. Additionally, in independent testing, the AUC values obtained from our method are significantly improved by 11.9% on four testing datasets averagely. It is noteworthy that the AUC values obtained by our method are all higher than PredBLP's method. Thus, all comparisons suggest that our proposed method is powerful and reliable for BLP identification.

### 3.7. Comparison of Identifying Novel BLPs in UniProt Database

Additionally, the computational tools should be used to identify novel and unknown proteins, which can provide convenient and accurate annotation. To examine the scalability and robustness of the reviewed predictors, we used another independent test data that were not applied in above analysis. Thus, we adopted the BLP data that were deposited from August 2016 to February 2017 in UniProt database. These novel BLP data collected by Zhang et al. [15] were derived from bacteria, eukaryote, and archaea. Then, based on the same novel BLP data, we compared our method iBLP and Zhang et al.'s predictor PredBLP [15]. The results of PredBLP were obtained from their report. As listed in Table 7, for general, bacteria, and archaea, our proposed method achieved Acc values of >0.960, which is better than those for PredBLP. Especially for archaea, our model can even correctly identify all novel BLPs. However, the result for eukaryote was unsatisfactory. The limited number of eukaryote BLPs for species-specific model training could be the reason that account for this.

## 4. Conclusions

Bioluminescent proteins (BLPs) are commonly exist in many living organisms, and identifying BLPs has significant importance for disease diagnosis and biomedical engineering. In this study, we proposed a novel predicting framework for the identification of BLPs by using sequence-derived features. To improve the prediction performance for BLPs, we examined the performance of several kinds of features and classification algorithms. Thus, based on the optimal feature subsets and XGBoost algorithm, we constructed an online predictor called iBLP. Given that very few webservers for BLP identification are still working, our webserver will be well maintained for two years or more. Besides, a software package for bioluminescent proteins identification in batch in users' local computers was developed and available at http://lin-group.cn/server/iBLP.

Experiments on benchmark datasets proved the robustness and effectiveness of our method. Moreover, the intrinsic properties of BLPs against non-BLPs have been analyzed in previous work, which reflected that it is necessary to distinguish various species of BLPs. Our experiments also demonstrated that BLP sequences have species specificity, suggesting that one should establish species-specific predictor. However, the benchmark datasets for eukaryote and archaea are not large enough, which may result in the bias for accuracy evaluation. In the future, with the accessibility of more BLP data, we will update the models by training them on large datasets. Additionally, we will take full consideration of difference of species-specific BLP data to select the majority of the informative features and establish more powerful and reliable models. We hope that our work can provide convenience to the experimental scientists to obtain the desired results rapidly and accurately without repeating the mathematical details.

## Figures and Tables

**Table 1:** The constructed benchmark datasets for BLP prediction.

Dataset	Group	Species			
		Bacteria	Eukaryote	Archaea	General
Training	Positive	598	56	36	690
	Negative	598	56	36	690
Testing	Positive	150	14	9	173
	Negative	4321	1370	712	6403

**Table 2:** Amino acid physicochemical attributes used in CTD method and the three corresponding groups of amino acids according to each attribute.

Attributes	Groups		
	I	II	III
Hydrophobicity_PRAM900101	RKEDQN	GASTPHY	CLVIMFW
Hydrophobicity_ARGP820101	QSTNGDE	RAHCKMV	LYPFIW
Hydrophobicity_ZIMJ680101	QNGSWTDERA	HMCKV	LPFYI
Hydrophobicity_PONP930101	KPDESNQT	GRHA	YMFWLCVI
Hydrophobicity_CASG920101	KDEQPSRNTG	AHYMLV	FIWC
Hydrophobicity_ENGD860101	RDKENQHYP	SGTAW	CVLIMF
Hydrophobicity_FASG890101	KERSQD	NTPG	AYHWVMFLIC
Normalized Van der Waals volume	GASTPDC	NVEQIL	MHKFRYW
Polarity	LIFWCMVY	PATGS	HQRKNED
Polarizability	GASDT	CPNVEQIL	KMHFRYW
Charge	KR	ANCQGHILMFPSTWYV	DE
Secondary structure	EALMQKRH	VIYCWFT	GNPSD
Solvent accessibility	ALFCGIVW	RKQEND	MSPTHY

**Table 3:** The published benchmark dataset for the prediction of BLPs.

Datasets	Year	Training set		Independent test set		Sequence Identity	Reference
		BLPs	Non-BLPs	BLPs	Non-BLPs		
D1	2011	300	300	141	18202	≤40% (CD-HIT)	[10]
	2012	300	300	139	18202	≤40% (CD-HIT)	[11]
	2013	199	199	141	137	≤40% (CD-HIT)	[12]
	2014	274	274	234	220	≤40% (CD-HIT)	[13]
	2015	441	13446	NA	NA	≤40% (CD-HIT)	[14]
D2	2017	863	7093	690	5674	≤30% (BlastClust)	[15]

^∗^NA denotes not applied.

**Table 4:** A comprehensive list of the reviewed methods/tools for the prediction of BLPs.

Tool^a^ (year)	Species	Feature representation	Feature selection	Classification algorithm	Work (yes/no)
BLPort [10] (2011)	NA	PCP	ReliefF	SVM	No
BLPre (2012)	NA	PSSM-AC	NA	SVM	No
Fan's method [12] (2013)	NA	DC+PSSM+acACS+RAAA	ID	SVM	NA
SCMBLP [13] (2014)	NA	DC	NA	SCM	No
Nath's method [14] (2015)	NA	AAC+AAGC+physicochemical n-grams	ReliefF	RARF	NA
PredBLP (2017)	Bacteria, eukaryote, archaea	AAC+dc+MTF+PCP	SBS	SVM	Yes

^∗^NA: not applied; PCP: physicochemical properties; PSSM-AC: position-specific scoring matrix and auto covariance; DC: dipeptide composition; acACS: auto covariance average chemical shift; AAC: amino acid composition; AAGC: amino acid property group composition; MTF: sequence motifs; ID: increment of diversity; SBS: sequential backward selection; SVM: support vector machine; SCM: scoring card method; RARF: Real Adaboosting Random Forest. ^a^The URL addresses for accessing the listed and available tools are provided as follows: BLPort: http://www.inb.uni-luebeck.de/tools-demos/bioluminescent%20protein/BLProt. BLPre: http://59.73.198.144/AFP_PSSM/. SCMBLP: http://iclab.life.nctu.edu.tw/SCMBLP/index.html.PredBLP: http://www.inforstation.com/webservers/PredBLP/predict.html.

**Table 5:** Performance evaluation on different features and combinations on four training datasets by 10-fold cross validation.

Features	General					Bacteria					Eukaryote					Archaea				
	Sn	Sp	Acc	MCC	AUC	Sn	Sp	Acc	MCC	AUC	Sn	Sp	Acc	MCC	AUC	Sn	Sp	Acc	MCC	AUC
NV	0.820	0.833	0.827	0.655	0.887	0.841	0.848	0.845	0.690	0.916	0.680	0.810	0.745	0.508	0.786	0.825	0.850	0.838	0.723	0.925
CTD	0.813	0.835	0.824	0.649	0.884	0.820	0.845	0.832	0.665	0.910	0.767	0.820	0.793	0.596	0.875	0.842	0.767	0.804	0.634	0.904
g-gap DC	0.786	0.849	0.817	0.637	0.892	0.798	0.876	0.837	0.678	0.910	**0.847**	**0.800**	**0.823**	**0.667**	**0.891**	**0.808**	**0.867**	**0.838**	**0.696**	**0.933**
PseAAC	**0.816**	**0.848**	**0.832**	**0.665**	**0.900**	**0.831**	**0.858**	**0.845**	**0.691**	**0.925**	0.743	0.713	0.728	0.469	0.810	0.808	0.825	0.817	0.650	0.904
NV+CTD	0.838	0.863	0.851	0.702	0.900	0.861	0.873	0.867	0.735	0.925	0.727	0.803	0.765	0.548	0.856	0.908	0.783	0.846	0.705	0.917
NV+g-gap DC	0.822	0.855	0.838	0.678	0.901	0.826	0.880	0.853	0.708	0.921	0.800	0.827	0.813	0.636	0.904	0.875	0.792	0.833	0.685	0.908
NV+PseAAC	0.846	0.859	0.853	0.707	0.911	0.840	0.876	0.858	0.717	0.932	0.720	0.823	0.772	0.554	0.848	0.800	0.808	0.804	0.637	0.926
CTD+g-gap DC	0.832	0.857	0.844	0.690	0.905	0.831	0.873	0.852	0.705	0.925	**0.857**	**0.840**	**0.848**	**0.710**	**0.924**	0.900	0.833	0.867	0.750	0.950
CTD+PseAAC	0.823	0.868	0.846	0.693	0.908	0.849	0.880	0.865	0.730	0.932	0.730	0.803	0.767	0.550	0.890	0.800	0.833	0.817	0.654	0.915
g-gap DC+PseAAC	0.799	0.870	0.834	0.670	0.906	0.839	0.880	0.860	0.721	0.934	0.810	0.857	0.834	0.682	0.903	0.883	0.808	0.846	0.707	0.934
NV+CTD+g-gap DC	0.842	0.859	0.851	0.703	0.906	0.851	0.873	0.862	0.726	0.930	0.777	0.840	0.808	0.629	0.904	**0.925**	**0.850**	**0.888**	**0.808**	**0.969**
NV+CTD+PseAAC	0.835	0.861	0.848	0.697	0.909	0.836	0.881	0.859	0.720	0.934	0.700	0.783	0.742	0.515	0.856	0.867	0.867	0.867	0.742	0.928
NV+g-gap DC+PseAAC	0.839	0.875	0.857	0.716	0.917	0.836	0.881	0.867	0.736	0.934	0.693	0.753	0.723	0.462	0.832	0.883	0.842	0.838	0.689	0.958
CTD+g-gap DC+PseAAC	0.829	0.874	0.851	0.705	0.916	**0.846**	**0.888**	**0.867**	**0.736**	**0.936**	0.800	0.823	0.812	0.644	0.910	0.892	0.775	0.833	0.688	0.934
NV+CTD+g-gap DC+PseAAC	**0.832**	**0.867**	**0.850**	**0.700**	**0.920**	0.845	0.878	0.861	0.724	0.936	0.803	0.783	0.793	0.625	0.903	0.892	0.775	0.833	0.688	0.958

^∗^ NV: natural vector method; CTD: composition, transition, and distribution; g-gap DC: g-gap dipeptide composition; PseAAC: pseudo amino acid composition. Besides, the bold marks the best result for individual and combined features, respectively.

**Table 6:** Comparison of our model with the existing method on the same datasets.

Method	General					Bacteria					Eukaryote					Archaea				
	Sn	Sp	Acc	MCC	AUC	Sn	Sp	Acc	MCC	AUC	Sn	Sp	Acc	MCC	AUC	Sn	Sp	Acc	MCC	AUC
PredBLP^1^	0.732	0.949	0.841	0.698	0.883	0.832	0.943	0.888	0.780	0.920	0.667	0.833	0.750	0.510	0.806	0.825	0.900	0.863	0.733	0.917
Our method^1^	0.835	0.877	0.856	0.713	0.915	0.841	0.885	0.863	0.727	0.932	0.823	0.911	0.867	0.744	0.939	0.889	0.779	0.834	0.675	0.934
PredBLP^2^	0.611	0.921	0.913	0.294	0.784	0.638	0.927	0.917	0.352	0.817	0.750	0.946	0.944	0.301	0.836	0.750	0.922	0.920	0.279	0.789
Our method^2^	0.867	0.885	0.884	0.352	0.942	0.873	0.895	0.894	0.411	0.942	0.643	0.899	0.896	0.175	0.849	1.000	0.837	0.839	0.246	0.964

**Table 7:** Comparison of iBLP with other methods on novel BLPs.

Species	Number	Method	Acc
General	3741	PredBLP	0.889
		Our method	0.963
Bacteria	3614	PredBLP	0.912
Eukaryote	106	Our method	0.962
		PredBLP	0.983
Archaea	21	Our method	0.708
		PredBLP	0.993
		Our method	1.000

## Data Availability

The data used to support the findings of this study are from previously reported studies and public database, which have been cited.
